# Involvement of the Postrhinal and Perirhinal Cortices in Microscale and Macroscale Visuospatial Information Encoding

**DOI:** 10.3389/fnbeh.2020.556645

**Published:** 2020-10-09

**Authors:** Nithya Sethumadhavan, Thu-Huong Hoang, Christina Strauch, Denise Manahan-Vaughan

**Affiliations:** ^1^Department of Neurophysiology, Medical Faculty, Ruhr University Bochum, Bochum, Germany; ^2^International Graduate School of Neuroscience, Ruhr University Bochum, Bochum, Germany

**Keywords:** postrhinal cortex, perirhinal cortex, fluorescence *in situ* hybridization, immediate early gene, visuospatial information processing

## Abstract

Whereas the postrhinal cortex (POR) is a critical center for the integration of egocentric and allocentric spatial information, the perirhinal cortex (PRC) plays an important role in the encoding of objects that supports spatial learning. The POR and PRC send afferents to the hippocampus, a structure that builds complex associative memories from the spatial experience. Hippocampal encoding of item-place experience is accompanied by the nuclear expression of immediate early gene (IEGs). Subfields of the Cornus ammonius and subregions of the hippocampus exhibit differentiated and distinct encoding responses, depending on whether the spatial location and relationships of large highly visible items (macroscale encoding) or small partially concealed items (microscale encoding), is learned. But to what extent the PRC and POR support hippocampal processing of different kinds of item-place representations is unclear. Using fluorescence *in situ* hybridization (FISH), we examined the effect of macroscale (overt, landmark) and microscale (subtle, discrete) item-place learning on the nuclear expression of the IEG, Arc. We observed an increase in Arc mRNA in the caudal part of PRC area 35 and the caudal part of the POR after macroscale, but not microscale item-place learning. The caudal part of PRC area 36, the rostral and middle parts of PRC areas 35 and 36, as well as the middle part of the POR responded to neither type of item. These results suggest that macroscale items may contain a strong identity component that is processed by specific compartments of the PRC and POR. In contrast small, microscale items are not encoded by the POR or PRC, indicating that item dimensions may play a role in the involvement of these structures in item processing.

## Introduction

The parahippocampal regions comprise the perirhinal cortex (PRC), postrhinal cortex (POR), and entorhinal cortex, along with the presubiculum and parasubiculum (Witter et al., 2017). Together, these regions serve as a gateway for unimodal and polymodal associational inputs directed to the hippocampal formation (Furtak et al., 2007). The two-streams hypothesis postulates that, in the parahippocampal regions, two functionally distinct information-processing streams exist: the dorsal stream is specialized in spatial memory (“where” stream) and the ventral stream in non-spatial memory (“what” stream; Ungerleider and Mishkin, 1982; Eichenbaum and Lipton, 2008). According to this hypothesis, spatial information is processed in the POR and sent to the hippocampus *via* the medial entorhinal cortex (MEC), whereas non-spatial information is processed by the PRC *via* the lateral entorhinal cortex (LEC) before the hippocampus is involved (Burwell et al., 1995, 2004; Burwell and Amaral, 1998b; Eichenbaum and Lipton, 2008). More recent studies suggest, however, that the functions of the parahippocampal regions do not strictly align to the two-streams hypothesis: *both* the POR and PRC are involved in different kinds of spatial and non-spatial information processing (Brown and Aggleton, 2001; Ramos, 2002, 2008, 2013a,b, 2017; Burwell et al., 2004; Winters et al., 2004; Ramos and Vaquero, 2005; Furtak et al., 2012; Heimer-McGinn et al., 2017; Ramos, 2017; Burke et al., 2018; LaChance et al., 2019).

Spatial learning and navigation rely on a multimodal cognitive map of the environment (Kelly et al., 2008) that represents allocentric spatial transformations, which encode information about the location of one object concerning other objects, and egocentric perspective transformations that reflect a self-object representational system (Ekstrom et al., 2014; Wang et al., 2020). A potential center for the integration of egocentric and allocentric spatial information in the rat brain is the POR: this structure projects strongly to the MEC that engages in the encoding of information of an allocentric spatial map (Burwell and Amaral, 1998b; Burwell, 2000; Wang et al., 2020). The POR also projects to the LEC, which is involved in the encoding of an egocentric spatial map and in object recognition memory (Burwell, 2000; Wang et al., 2018; Doan et al., 2019; LaChance et al., 2019). These studies support that the POR may act as a conduit for spatial information processing directed to both entorhinal subdivisions (Winters et al., 2004; LaChance et al., 2019). Besides these properties, the POR is also reciprocally connected with visual associational cortices (Burwell and Amaral, 1998a; Agster and Burwell, 2009; Agster et al., 2016) and it supports visual object discrimination (Furtak et al., 2012). Thus, the POR is involved in the processing of both spatial and non-spatial information (Furtak et al., 2012; Heimer-McGinn et al., 2017; Ramos, 2017; Burke et al., 2018).

The PRC plays a role in object recognition memory (Brown and Aggleton, 2001). It has been proposed that it combines different sensory features of the object, which means object identification is facilitated (Eacott et al., 1994, 2001; Murray and Bussey, 1999; Murray et al., 2007). Moreover, this region is involved in ambiguous-feature discrimination learning (Bussey et al., 1999, 2000; Eacott et al., 2001; Eacott and Norman, 2004; Murray et al., 2007), suggesting that it also supports more subtle aspects of item differentiation. The PRC sends an indirect anatomical projection to the hippocampus *via* the LEC (Burwell and Amaral, 1998a,b) and also sends afferents directly to the CA1 region and the subiculum (Agster and Burwell, 2013). Furthermore, it strongly projects to frontal and other neocortical associational regions (Burwell and Amaral, 1998a,b; Burwell, 2000; Kealy and Commins, 2011). In addition, the PRC receives afferent input from the POR (Burwell and Amaral, 1998a,b; Furtak et al., 2007; Agster and Burwell, 2009). Thus, the PRC is well-positioned to provide information about items and other discrete stimuli to the hippocampus for memory formation, and to neocortical regions for other cognitive functions (Brown and Aggleton, 2001; Kealy and Commins, 2011). Moderate projections of the PRC to thalamic structures suggest that the PRC may also support crossmodal, perceptual, and attentional processing (van der Werf et al., 2002) and results from lesion studies in monkeys indicate that the PRC is necessary for identifying visual novelty (Meunier et al., 1993).

The PRC and LEC are involved in non-spatial information processing (Burwell et al., 1995, 2004; Zhu et al., 1995a,b; Burwell and Amaral, 1998a,b; Hargreaves et al., 2005; Deshmukh et al., 2012). However, the involvement of the PRC in spatial information processing is supported by several studies: In particular, an engagement of the PRC in allocentric spatial learning has been demonstrated (Ramos, 2002, 2008, 2013a,b, 2017; Aggleton et al., 2004; Burwell et al., 2004; Ramos and Vaquero, 2005; Futter et al., 2006). Moreover, it has been reported that lesioning the PRC causes task-learning deficits in the acquisition and retrieval phase of spatial working memory (Meunier et al., 1993; Maioli et al., 2012). Anatomical studies have shown that the PRC can be subdivided into areas 35 and 36 (Brodmann, 1909; Burwell et al., 1995). PRC area 36 occupies the dorsal side and area 35 occupies the ventral side of the rhinal sulcus (Burwell, 2001). Their specific roles in the processing of spatial memory are still unclear.

Previous studies have revealed that different forms of item-place learning elicit potent and differentiated effects on hippocampal information processing: novel exposure to discretely placed visuo-, audio- or olfacto-spatial (microscale) cues that can only be detected when the animal is close to them facilitates long-term depression (LTD) in the CA1 and CA3 regions of the hippocampus (Kemp and Manahan-Vaughan, 2004, 2008; André and Manahan-Vaughan, 2013; Dietz and Manahan-Vaughan, 2017). By contrast, novel exposure to large, highly visible landmark (macroscale) cues, that can be seen from afar, facilitates input-specific LTD in the dorsal dentate gyrus (DG; Kemp and Manahan-Vaughan, 2008) and CA3 region (Hagena and Manahan-Vaughan, 2011). Regardless of the hippocampal region and whether micro- or macroscale cues are presented, a second exposure to the cues in their previous spatial positions does not facilitate LTD anew, whereas a new spatial configuration of the items enables *de novo* LTD (Manahan-Vaughan and Braunewell, 1999; Kemp and Manahan-Vaughan, 2004, 2008; Hagena and Manahan-Vaughan, 2011). Novel microscale item-place learning also triggers neuronal encoding in the form of nuclear immediate early gene (IEG) expression: an increase in nuclear IEG expression in the distal CA1 region and proximal CA3 region occurs following microscale item-place learning, whereas novel exposure to macroscale cue configurations results in an increase in nuclear IEG expression in the lower blade of the DG and the proximal CA3 region (Hoang et al., 2018). These findings indicate that: (1) the enabling of synaptic plasticity by item-place exploration is tightly associated with the learning of the spatial location of the items; (2) hippocampal processing of allocentric item information is conducted in a subfield-specific manner that is determined by the spatial content; and (3) item context and/or item dimensions in allocentric space is differentiated during spatial information storage by the hippocampus. Thus, within the hippocampus a clear division of labor is evident in terms of the functional read-outs of macroscale and microscale spatial cue encoding in hippocampal subfields. This raises the question as to whether the hippocampal is supported in these forms of differentiated information processing by parahippocampal regions, and if so, whether functional discrimination also occurs in candidate regions, such as the PRC and POR.

In the present study, we, therefore, explored to what extent POR and PRC are involved in the distinction between microscale and macroscale item-place processing. We used fluorescence *in situ* hybridization (FISH) to map activity-dependent mRNA expression of the IEG Arc in neurons of the POR and PRC, following microscale and macroscale item-place learning. We observed an increase in Arc mRNA expression in the nuclei of neurons in both area 35 of the caudal part of the PRC, as well as the caudal POR after the exploration of macroscale cues, but not after microscale cue exploration. By contrast, no significant changes in Arc mRNA expression were detected in nuclei of neurons in area 36 of the caudal part of the PRC, the rostral and middle part of PRC areas 35 and 36, or the middle compartment of the POR after exploration of the macroscale or microscale cues. These results support that area 35 of the PRC and caudal POR, are involved in macroscale visuospatial information processing, thereby indicating that these regions can functionally discriminate between different item dimensions and/or context.

## Materials and Methods

This study was carried out following the European Communities Council directive of 22 September 2010 (2010/63/EU) for the care of laboratory animals, and all experiments were conducted after approval of the ethics committee of the federal government of the state of North Rhine-Westphalia (NRW; Landesamt für Arbeitsschutz, Naturschutz, Umweltschutz und Verbraucherschutz, NRW). All efforts were made to reduce the number of animals used.

Seven- to 9-week-old male adult Wistar rats were used for the study, using a behavioral paradigm that was previously established by our group (Kemp and Manahan-Vaughan, 2004; Hoang et al., 2018). The animals were housed in an animal housing facility in temperature (22 ± 2°C) and humidity (55 ± 5%) monitored containers (Scantainer, Scanbur Technology A/S, Karlslunde, Denmark) on a 12 h light/12 h dark cycle (lights on from 7 a.m. to 7 p.m.). Animals had *ad libitum* access to water and food.

### Novel Item-Place Exploration Task

Novel exploration tasks were performed as described previously (Hoang et al., 2018). In brief, experiments, including habituation, were conducted in gray chambers (washable polyvinyl chloride, 40 cm width × 40 cm length × 50 cm height, open at the top, translucent, and removable front wall, illumination of experimental room: ca. 500 lux). On two consecutive days before the final experiment (test day), animals were handled by the experimenter for 15 min and habituated to the recording chamber for 1 h per day. After the habituation, animals were returned to their home cages. On the test day, animals were again habituated to the same chamber. They resided in the recording chamber for 1 h until the learning tasks were commenced.

#### Microscale Item-Place Exploration (Figure 1A)

After the final habituation phase on the test day, animals explored a holeboard that was inserted onto the floor of the familiar chamber. The holeboard (39.8 cm width × 39.8 cm length × 5 cm height, gray washable polyvinyl chloride) contained four holes (5.5 cm in diameter, 5 cm depth) that were equidistant (1 cm) from its edge. One of three visually different small objects (ca. 2 cm width × 2 cm length × 4 cm height) was placed inside three of the four holes. These objects did not extend above the surface of the holeboard. Animals could not see the objects from afar and had to approach the holes and put their noses inside them to explore the objects. This task facilitates LTD in the CA1 and CA3 regions of the hippocampus (Manahan-Vaughan and Braunewell, 1999; Kemp and Manahan-Vaughan, 2004, 2008; Hagena and Manahan-Vaughan, 2011) and elevates nuclear Arc mRNA expression in the hippocampus of rats (Hoang et al., 2018). Five minutes after the exploration started, animals’ brains were quickly collected.

#### Macroscale Item-Place Exploration (Figure 1B)

On the test day, three large landmark objects (object 1: 10 × 8 × 7 cm width × length × height, object 2: 6 cm × 11 cm, and object 3: 8 × 10 cm, diameter × height) were placed on the floor of the recording chamber, after the final habituation phase. The objects were placed at a minimum distance of 3 cm from the walls of the chamber so that the animals could circle them if they so wished. These landmark objects serve as directional cues for navigation and can be viewed from afar. Previous studies have shown that this task induces LTD in the CA3 region and DG of Wistar rats (Kemp and Manahan-Vaughan, 2004, 2008; Hagena and Manahan-Vaughan, 2011). Furthermore, exposure to these landmark objects triggers a significant increase in nuclear IEG expression in the CA3 region and DG (Hoang et al., 2018). Animals explored the objects for 5 min and directly after the end of the exploration, brains were quickly removed.

Control animals underwent the same handling and habituation procedure as both test groups. On the test day, after the habituation phase inside the chamber, brains were extracted.

### Fluorescence *in situ* Hybridization (FISH)

We conducted FISH to detect the nuclear expression of Arc mRNA. The Arc gene (also known as Arg3.1) is an effector IEG (Link et al., 1995; Lyford et al., 1995; Brakeman et al., 1997) and its expression can be induced by neural activity (Abraham et al., 1993; Worley et al., 1993) and behavioral training (Hess et al., 1995; Vann et al., 2000).

For FISH, brains were rapidly removed (within 2 min) and shock-frozen in 2-methyl butane at −80°C. Later, 20 μm thick coronal sections (three sections per glass slide) containing the PRC (from ca. −2.7 to −5.8 mm posterior to Bregma) and the POR (ca. −6.6 to −8.1 mm posterior to Bregma; Paxinos and Watson, 2005) were cut on a Cryostat (Leica CM 3050S), mounted directly on glass slides (SuperFrost Plus, Gerhard Menzel GmbH, Braunschweig, Germany) and stored at −80°C until further processing. The differentiation of the PRC and POR into compartments was based on the methodology used by others (Burwell, 2001; Furtak et al., 2007; Albasser et al., 2010, 2013; Agster and Burwell, 2013). Area 35 and 36 of the PRC were analyzed for the rostral (ca. −2.76 to −3.84 mm posterior to Bregma), middle (ca −3.84 to −4.80 mm posterior to Bregma), and caudal PRC (ca. −5.16 to −5.76 mm posterior to Bregma; Albasser et al., 2010, 2013). For the POR, the middle (ca. −6.6 to −7.08 mm posterior to Bregma) and caudal components (ca. −7.2 to −8.04 mm posterior to Bregma) were analyzed (Agster and Burwell, 2009; Kinnavane et al., 2016; Burwell, 2001).

Arc cDNA plasmid (Entelechon, Bad Abbach, Germany) was prepared using a 3 kb Arc transcript according to the sequence of Lyford et al. (1995). The cRNA probes were prepared from the linearized cDNA using Ambion MaxiScript Kit (Invitrogen, Carlsberg, CA, USA) and a premixed RNA labeling nucleotide mix containing digoxigenin-labeled UTP (Invitrogen, Waltham, MA, USA). After purification on Mini Quick SpinRNA columns (Roche Diagnostics, Mannheim, Germany) we verified the yield and integrity of the RNA probes using gel electrophoresis.

For FISH, one slide per animal (with three sections each) was chosen and left at room temperature (RT) for 1 h. From each animal, the slide with sections for the rostral PRC at ca. −3.24 mm, middle PRC at ca. −4.56 mm, caudal PRC at ca. −5.52 mm, middle POR at ca. −6.96 mm, and caudal POR at ca. −7.8 mm posterior from Bregma was chosen (Paxinos and Watson, 2005). FISH for digoxigenin-labeled Arc was performed as described previously (Hoang et al., 2018), adapted from (Guzowski et al., 1999). For this, slides were fixed in ice-cold 4% paraformaldehyde in phosphate-buffered saline (PBS) for 5 min and then washed in 2× saline sodium citrate buffer (SSC) for 2 min. The slides were left in acetic anhydride solution for 10 min, quickly washed five times each 1 min in diethylpyrocarbonate (DEPC)-treated water, and left in 2× SSC finally for 5 min. The humidity chamber was prepared with 2× SSC/50 deionized formamide (Sigma–Aldrich, St. Louis, MO, USA; a/a) soaked filter paper. 100 μl of 1× prehybridization buffer (Sigma–Aldrich, St. Louis, MO, USA) was applied on each slide for 30 min at RT. The slides were covered with a piece of laboratory film (Parafilm^®^, Bemis, Neenah, WI, USA) to prevent the brain material from drying out. The fluorescein-labeled DNA probe was diluted at a concentration of 1 ng/1 μl in a 1× hybridization buffer (Sigma–Aldrich, St. Louis, MO, USA), heated at 90°C for 5 min and distributed on the slides. The slides were again covered with laboratory film (Parafilm^®^, Bemis, Neenah, WI, USA) and hybridized for approximately 17 h in a humid chamber at 56°C. Following the hybridization, the stringent washing steps were conducted. Laboratory film (Parafilm^®^, Bemis, Neenah, WI, USA) was removed and the slides were placed in 2× SSC at 56°C thrice for 5 min each. RNase (Sigma–Aldrich, St. Louis, MO, USA) was dissolved in 2× SSC to a concentration of 100 μg/100 ml 2× SSC and stored at 37°C. The slides were placed into the RNase-solution for 15 min at 37°C, followed by a 10 min 2× SSC buffer at 37°C. Stringent washing was continued in 0.5× SSC for 10 min at 56°C, 0.5× SSC for 30 min at 56°C, 0.5× SSC for 10 min at RT, hydrogen peroxidase (30% H_2_O_2_ in 1× SSC) for 15 min, three times 5 min in 1× SSC at RT and finally rinsed in tris-buffered saline (TBS) at RT for 5 min. Signal detection was carried out by immunohistochemistry. Arc-digoxigenin was detected by Anti-digoxigenin (Roche Holding AG, Basel, Switzerland) in 1% n-goat serum and 100 μl TBS for 120 min. The slides were then washed three times each 5 min in TBS-Tween. The Arc mRNA signal was visualized using Streptavidin Cy5 (Dianova, Hamburg, Germany), then the slides were washed with TBS Tween for 5 min. After slides were rinsed in TBS, they were washed in distilled water and dipped in 70% ethanol several times. The sections were stained using 1% Sudan black B (Sigma–Aldrich, St. Louis, MO, USA) in 70% ethanol (Oliveira et al., 2010). Then, slides were washed with distilled water and air-dried overnight. The nuclei were visualized using 4′,6-diamidino-2-phenylindole (DAPI) in mounting medium (Cat No. SCR: 38448, Dianova, Hamburg, Germany).

### Image Acquisition

Arc mRNA expression was examined within the nuclei of pyramidal and non-pyramidal cells in layer 2 of the PRC and POR. *Z*-stacks were obtained at 63× magnification using a fluorescence microscope (Zeiss ApoTome) that permits structured illumination microscopy (Schaefer et al., 2004). For each region, three *z-stacks* from three consecutive slices were obtained for each animal.

### Data Analysis

For the analysis of the behavioral data, the total exploration time (of the total exposure time of 5 min) inside the chamber was determined for each animal. Here, exploration was defined as all behaviors excluding resting, sleeping, and grooming. Furthermore, the exploration time of each of the three objects or each of the four holes was determined, to ensure that microscale and macroscale cues were explored (no exploration of a hole or object was set as an exclusion criterion for further analysis). The exploration time of the objects inside the holes of a holeboard was defined as the sum of all times the animals dipped its nose into each specific hole. For the exploration times of the macroscale cues, the animals’ nose had to be directed to the object as close as <l cm or touching the object for at least 2 s. For each of the three objects, the sum of these times was calculated. Also, the number of rears in the chamber was counted. A rear was defined as an event where the animal rose onto its hind paws. The front paws had either no contact with a wall, or one or both paws made contact with the wall or (during macroscale cue exploration) a landmark object, to support rearing.

The total exploration time and the exploration time per hole or object are calculated in seconds (s) ± standard error of the mean (SEM). Datasets were verified for normal distribution using the Kolmogorov–Smirnov test. For statistical analysis of the exploration time per object one-way ANOVA was performed with subsequent *post hoc* (Fisher’s LSD) analysis (Statistica, TIBCO Software Inc., Palo Alto, CA, USA). The total exploration time inside the chamber and the number of rears during microscale and macroscale cue exploration were compared and analyzed using one-way ANOVA. *Post hoc* values in the results section are only included if they revealed a significant result.

For the analysis of Arc mRNA expression in layer 2 of the POR and PRC compartments, complete nuclei were chosen and Arc positive signals were identified by examining each *z*-stack using ImageJ software (Schindelin et al., 2012). During “experimenter-blind” analysis, nuclei that contain Arc mRNA signal were counted and the percentage of Arc positive nuclei from all nuclei was calculated separately for each *z*-stack. Nuclei were analyzed in each *z-stack* of all regions and groups of the rostral PRC [21 ± 2.8 SD (standard deviation)], the middle PRC (20 ± 4.08 SD), the caudal (15 ± 2.77 SD), the middle POR (19 ± 4.10 SD) and the caudal POR (22 ± 3.82 SD). For each animal, the mean of the percentage of Arc mRNA expression of three *z*-stacks, for each region, was used for further analysis. For each region, the mean percentage ± SEM of all animals of a group (microscale cues, macroscale cues, or control) was calculated. The normal distribution of datasets of PRC and POR was confirmed using the Kolmogorov–Smirnov test. For statistical analysis of Arc mRNA expression, one-way ANOVA with subsequent *post hoc* analysis (Fisher’s LSD test) was performed to examine for each region differences elicited by microscale and macroscale item-place exploration compared to control animals. *Post hoc* values are only included in the results section if they revealed a significant difference between groups.

## Results

### Macroscale and Microscale Item-Place Exploration

**Figure 1:**
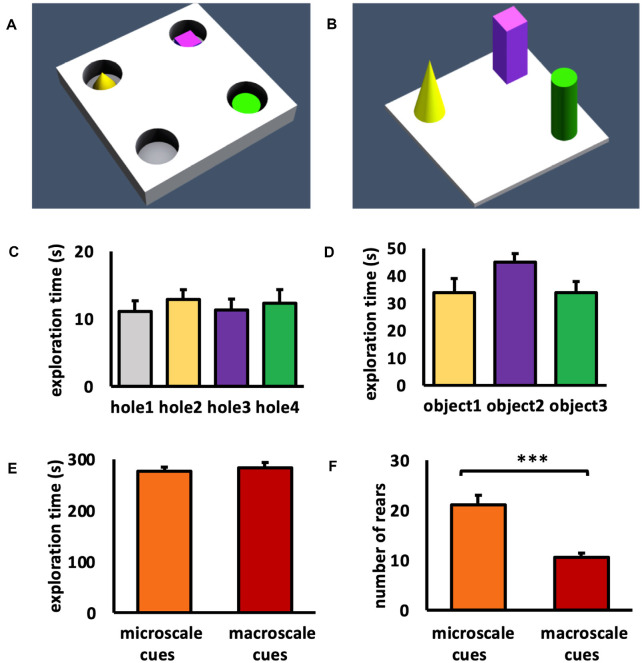
Schema of item-place configurations and object exploration behavior. **(A,B)** Animals either explored **(A)** three small novel objects (microscale cues) that were placed within three of four holes of a holeboard, or **(B)** three large novel objects (macroscale cues) that were placed on the floor of the chamber, for 5 min. **(C)** During microscale item-place exploration, the animals explored holes 2, 3, and 4 that contained microscale cues and hole 1 that did not contain an object, equally. ANOVA *F*_(3,60)_ = 0.2514, *p* = 0.860057. **(D)** During macroscale item-place exploration, no preference was evident concerning the time spent exploring objects 1, 2, and 3. ANOVA *F*_(2,36)_ = 1.9840, *p* = 0.152276. **(E)** No difference was evident between the total exploration times inside the chamber during microscale or macroscale item-place exposure. ANOVA *F*_(1,27)_ = 0.000, *p* = 0.98764. **(F)** Animals that explored microscale item-place configurations engaged in a significantly higher number of rears compared to animals that were exposed to macroscale item-place configurations. ANOVA *F*_(1,27)_ = 24.8538, *p* = 0.000032. **(C–F)** Mean ± SEM. **(F)** ANOVA: ***p < 0.001.

This study aimed to clarify whether the perirhinal and postrhinal cortices engage in the processing and encoding of novel microscale and macroscale item-place information (Figures 1A,B). Animals explored three small novel items (microscale cues) that were placed within three of four holes of a holeboard (Figure 1A), or three large novel objects (macroscale cues) that were placed on the floor of the chamber (Figure 1B). We refer to these tasks as macroscale (large, overt) and microscale (small, discrete) item-place learning, respectively, and compared effects of sizes of the cues (large vs. small) and their presentation strategy (overt vs. discrete) in item-place learning.

During microscale cue exploration (Figure 1C) we observed that the animals did not show item preference between holes 2, 3, and 4 that contained microscale cues and hole 1, which did not contain an object (one-way ANOVA: *F*_(3,60)_ = 0.2514, *p* = 0.860057). When the animals explored the three macroscale cues (Figure 1D), they also did not exhibit a preference for an individual object (one-way ANOVA: *F*_(2,36)_ = 1.9840, *p* = 0.152276). These results confirm that the animals did not show an item or hole bias during cue exposure.

To compare the exploration between the two test conditions, the total exploration time of the chamber during exposure to the microscale or macroscale cues was examined (Figure 1E). The total exploration time during exposure to both test conditions was equivalent (one-way ANOVA: *F*_(1,27)_ = 0.000, *p* = 0.987640). Furthermore, the number of rears during the 5 min exposure to micro- and macroscale item-place configurations was examined (Figure 1F). Animals that explored microscale cues showed a significantly higher number of rears, compared to animals that were exposed to macroscale cues (one-way ANOVA: *F*_(1,27)_ = 24.8538, *p* = 0.000032). This difference in rears may reflect the fact that whereas macroscale cues could be seen from afar and thus readily placed within a proximal and distal visual reference frame, microscale cues could only be seen when the animals poked their noses into the holes, requiring more effort to build a reference frame within a proximo-distal visuospatial context.

### Exploration of a Novel Microscale or Macroscale Item-Place Configuration Does Not Trigger Immediate Early Gene Expression in Areas 35 and 36 of the Rostral and Middle PRC

**Figure 2:**
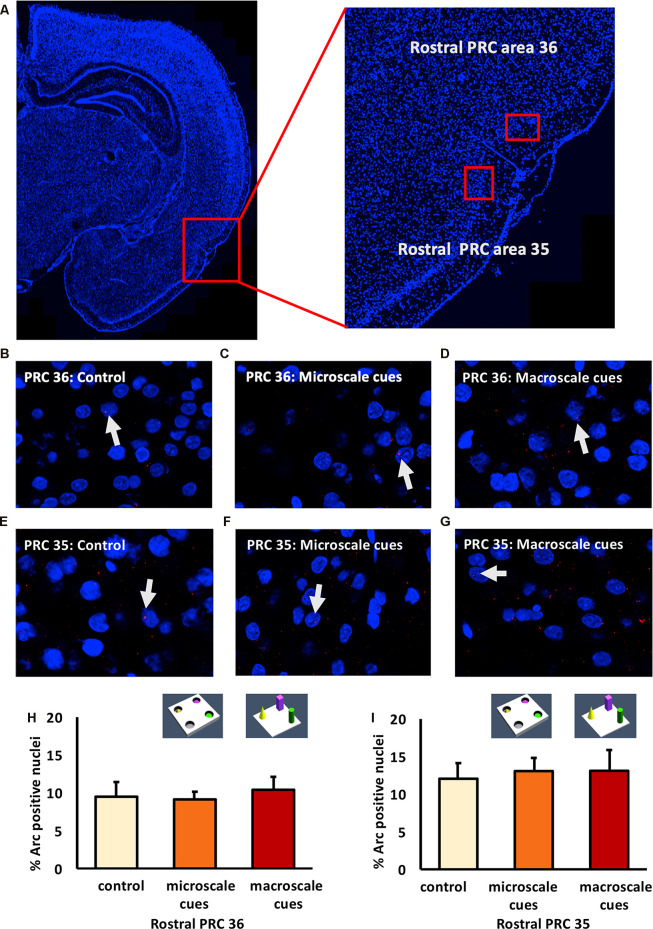
Exploration of item-place configurations does not trigger immediate early gene (IEG) expression in the rostral part of the perirhinal cortex (PRC). **(A)** Illustration of the rostral part of the PRC (outlined by a red square) in a DAPI-stained coronal section (ca. −3.24 mm posterior to Bregma) of the rat brain (left) and enlargement of the outlined area to show the rostral PRC (right) in which outlines of area 36 (top) and 35 (bottom) are included (indicated by red rectangles). **(B–G)** Representative images of Arc mRNA expression (red dots, indicated by white arrows) in rostral PRC area 36 **(B–D)** and area 35 **(E–G)** after novel exploration of microscale **(C,F)**, or macroscale **(D,G)** item-place configurations, compared to controls **(B,E)**. Images were taken using a 63× objective. Nuclei were stained with DAPI (blue). **(H,I)** Bar charts show the relative percentage of Arc-positive nuclei in the rostral PRC area 36 **(H)** and area 35 **(I)** following item-place exploration, compared to controls (mean ± SEM). Nuclear Arc mRNA expression in rostral PRC area 36 and area 35 remained unchanged after novel item-place exploration compared to control animals (ANOVA; area 36: *F*_(2,20)_ = 0.0756, *p* = 0.927454; area 35: *F*_(2,19)_ = 0.13803, *p* = 0.871941).

**Figure 3:**
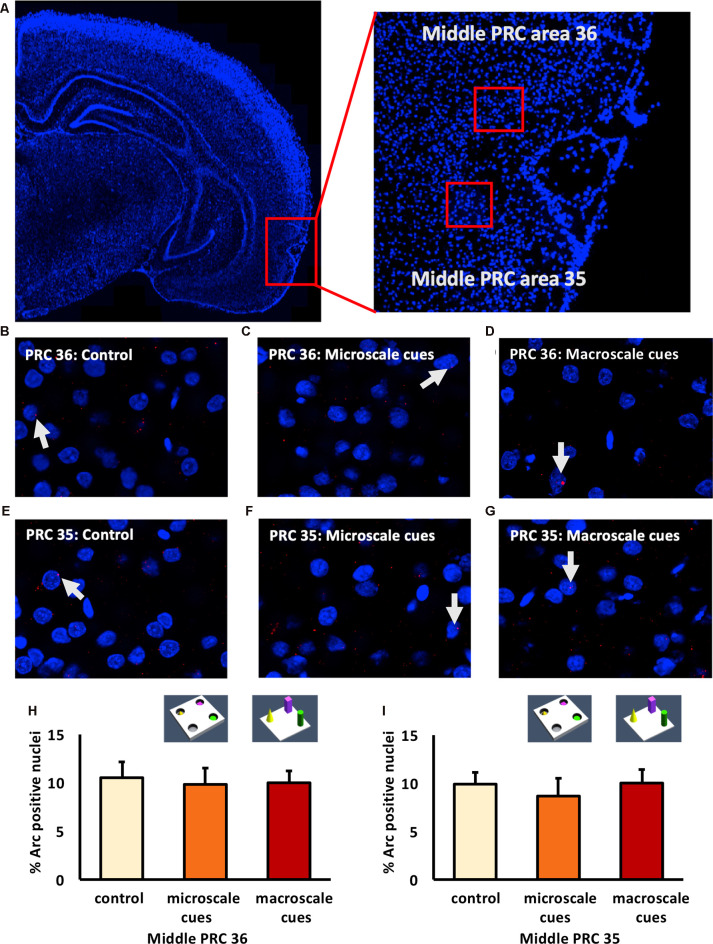
IEG expression in the middle part of perirhinal cortex area 35 and area 36 is unaffected by exposure to novel item-place configurations. **(A)** The left panel shows a DAPI-stained coronal section (ca. −4.56 mm posterior to Bregma) of the rat brain and the outline of the middle PRC (red square). The right panel shows an enlargement of the outlined area to show the middle PRC in which outlines of area 36 and 35 (red squares) are indicated, where *z*-stacks were taken for analysis. **(B–G)** Representative images of nuclear Arc mRNA positive nuclei (red dots, indicated by white arrows) in the middle PRC, area 36 **(B–D)** and PRC area 35 **(E–G)** following exploration of microscale cues **(C,F)**, or macroscale cues **(D,G)** compared to controls **(B,E)**. Blue: nuclear counterstaining with DAPI. Images were obtained using a 63× objective. **(H,I)** Bar charts describe the relative percentage of nuclei expressing Arc mRNA in the middle PRC after novel item-place exploration, compared to responses detected in control animals (mean ± SEM). No significant changes in nuclear Arc mRNA expression occured in PRC area 36 **(H)** or area 35 **(I)** in both experimental conditions, compared to controls (ANOVA; middle PRC area 36 *F*_(2,19)_ = 0.0630, *p* = 0.939180; middle PRC area 35 *F*_(2,19)_= 0.2444, *p* = 0.785604).

**Figure 4:**
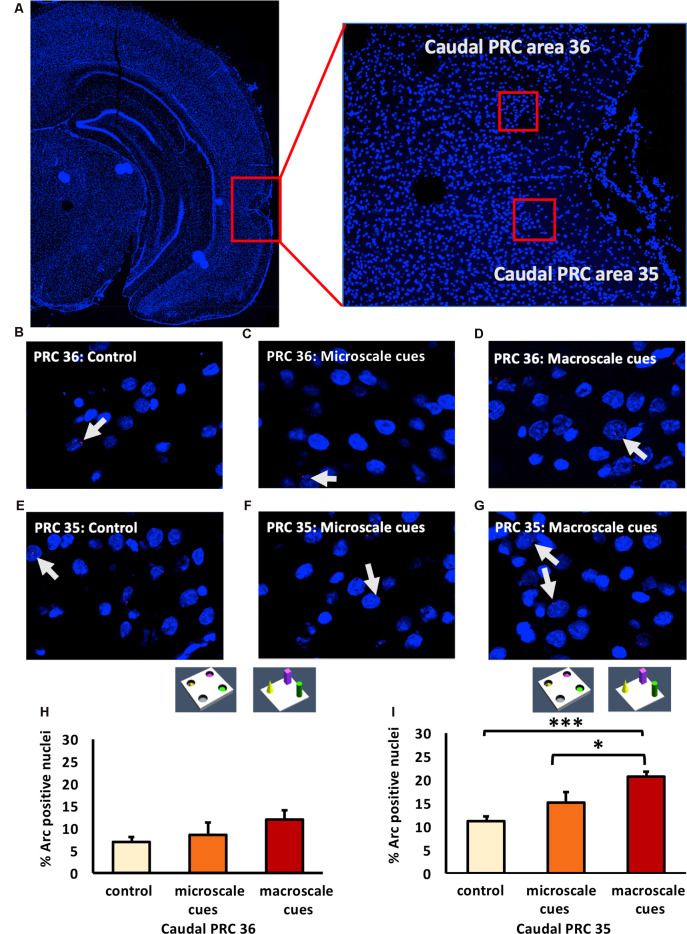
Exposure to a novel macroscale item-place configuration induces an increase in IEG expression in the caudal part of perirhinal cortex area 35, but not in area 36. **(A)** The left panel shows a DAPI-stained coronal section (ca. −5.52 mm posterior to Bregma) of the rat brain with the caudal PRC highlighted by a red rectangle. The right panel shows a magnification of the caudal PRC area. *Z*-stacks were created in the caudal PRC area 36 and 35, indicated by red squares. **(B–G)** Photomicrographs, taken using a 63× objective, show nuclear Arc mRNA expression (red points, indicated by white arrows) in the caudal PRC **(B–D)** area 36 and **(E–G)** area 35 following **(C,F)** microscale and **(D,G)** macroscale item-place exploration, or IEG expression in corresponding brain sections from control animals (control; **B,E**). Cell nuclei (blue) are stained with DAPI. **(H–I)** Bar charts represent the mean percentage (mean ± SEM) of Arc mRNA positive nuclei in caudal PRC area 36 **(H)** or PRC area 35 **(I)** under control or test conditions (ANOVA; caudal PRC area 35: *F*_(2,21)_ = 7.9498, *p* = 0.002689; area 36: *F*_(2,20)_ = 1.18071, *p* = 0.327571). Exploration of microscale item-place cues does not lead to significant changes in Arc mRNA expression in caudal PRC areas 35 and 36, relative to controls (microscale vs. control, *post hoc* Fisher’s LSD test, *p* > 0.05, each). Caudal PRC area 35, but not area 36, responds to macroscale item-place information (*post hoc* Fisher’s LSD test; area 36 *p* > 0.05; area 35: ***p < 0.001 for macroscale vs. control and *p < 0.05 for macroscale vs. microscale).

We previously reported that exploration of novel item-place constellations significantly elevated Arc mRNA expression in distinct subregions of the hippocampus (Hoang et al., 2018). Given the fact that the PRC sends direct and indirect anatomical projections to the hippocampus (Burwell and Amaral, 1998b; Agster and Burwell, 2009, 2013), we analyzed nuclear Arc mRNA levels following the same experimental conditions along the anterior-posterior axis of the PRC (Figures 2–4).

In area 36 of the rostral PRC (Figures 2B–D,H), novel exploration of microscale (*n* = 8) and macroscale (*n* = 7) item-place configurations did not change nuclear Arc mRNA expression (one-way ANOVA: *F*_(2,20)_ = 0.0756, *p* = 0.927454) compared to controls (*n* = 8). A similar result was detected for area 35 of the rostral PRC (Figures 2E–G,I). Here, too, IEG expression remained unchanged (one-way ANOVA: *F*_(2,19)_ = 0.13803, *p* = 0.871941) in animals that explored microscale and macroscale item-place configurations (*n* = 7, each) compared to controls (*n* = 8).

Examination of areas 35 and 36 of the middle part of the PRC (Figure 3) revealed that novel item-place learning has no influence on IEG expression in these regions. Compared to controls (*n* = 8, each) microscale (*n* = 7, each) and macroscale (*n* = 7, each) item-place learning did not change Arc mRNA levels in area 35 (Figures 3E–G,I; one-way ANOVA: *F*_(2,19)_ = 0.2444, *p* = 0.785604) and area 36 (Figures 3B–D,H; one-way ANOVA: *F*_(2,19)_ = 0.063, *p* = 0.93918) of the middle PRC. These findings suggest that areas 35 and 36 of the rostral and middle part of the PRC are not involved in microscale and macroscale visuospatial information processing.

### Nuclear mRNA Expression of Arc in Area 36 of the Caudal PRC Remains Unchanged Upon Exposure to Novel Item-Place Configurations

Examination of the caudal PRC revealed that exploration of a novel microscale (*n* = 8) or macroscale (*n* = 7) item-place configuration did not change IEG expression in the caudal PRC area 36 (Figures 4B–D,H; one-way ANOVA: *F*_(2,20)_ = 1.18071, *p* = 0.327571) compared to controls (*n* = 8). This result indicates that the caudal PRC area 36 is not involved in this kind of microscale and macroscale visuospatial information processing.

### Exposure to a Novel Macroscale Item-Place Configuration Triggers Nuclear Arc mRNA Expression in the Caudal PRC Area 35

In contrast, to the caudal PRC area 36, the caudal PRC area 35 engages in macroscale item-place learning (Figures 4E–G,I). Arc mRNA expression was significantly increased in the PRC area 35 of animals that explored macroscale item-place configurations, compared to control animals (*n* = 8, each; one-way ANOVA *F*_(2,21)_ = 7.9498, *p* = 0.002689). Exposure to microscale cues did not result in any significant differences in Arc mRNA expression, however, compared to the control group (*n* = 8, each post hoc Fisher’s LSD test *p* = 0.498398). Furthermore, the *post hoc* analysis revealed that the Arc mRNA expression level was significantly higher in animals that explored a novel macroscale item-place configuration compared to controls (Fisher’s LSD test: *p* = 0.000865) and to animals that explored a microscale item-place configuration (Fisher’s LSD test: *p* = 0.012388).

### Exploration of Novel Item-Place Configurations Do Not Change Nuclear Arc mRNA Expression in the Middle POR

**Figure 5:**
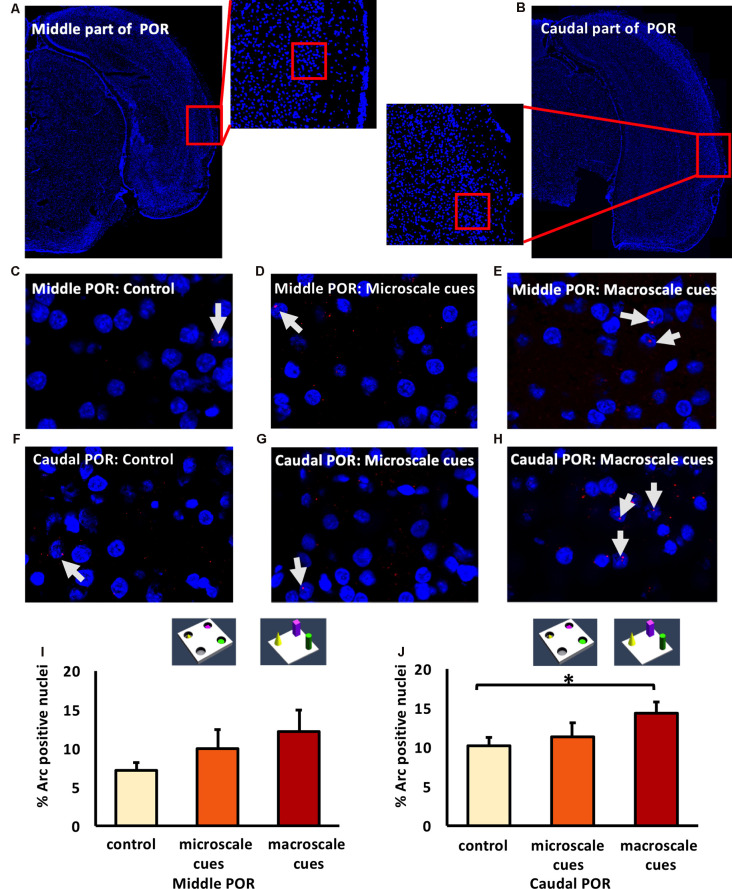
Differentiated IEG expression in the middle and caudal parts of the postrhinal cortex (POR) following the exploration of novel item-place configurations. **(A,B)** DAPI-stained coronal sections of the rat brain show the regions of interest examined in the **(A)** middle and **(B)** caudal parts of the POR, as indicated by red squares and the respective magnified images (middle). The red squares in enlarged images indicate areas where *z*-stacks were obtained for the middle POR (ca. −6.96 mm posterior to Bregma) and for the caudal POR (ca. −7.8 mm posterior to Bregma). **(C–H)** Photomicrographs represent nuclear Arc mRNA expression (red dots, indicated by white arrows) in the middle POR **(C–E)** and caudal POR **(F–H)** of control animals **(C,F)** or animals that participated in microscale **(D,G)** or macroscale item-place exploration **(E,H)**. Nuclei (blue) were stained with DAPI. Images were taken using a 63× objective. **(I,J)** Bar charts showing the relative percentage of positive Arc mRNA nuclei in the middle POR **(I)** and caudal POR **(J)** of controls and for both experimental conditions (mean ± SEM). No significant changes can be observed in the middle POR **(I)** following microscale and macroscale item-place exploration compared to the control group (ANOVA *F*_(2,19)_ = 1.19376, *p* = 0.324814). Interestingly, in caudal POR **(J)**, a significant difference in Arc mRNA expression can be detected in animals that participated in macroscale item-place exploration compared to the control group (*post hoc* Fisher’s LSD test, *p < 0.05), whereas exposure to microscale cues does not change Arc mRNA expression (*p* > 0.05).

The POR projects to the LEC and MEC, which in turn project to the hippocampus (Kerr et al., 2007; Agster and Burwell, 2013). We, therefore, explored whether the POR is involved in the processing of information about microscale and macroscale item-place configurations. We examined nuclear Arc mRNA expression in the middle and caudal POR following microscale (*n* = 8, each) and macroscale (*n* = 7, each) visuospatial learning (Figures 5A,B). In the middle POR nuclear Arc mRNA expression remained unchanged for both types of item-place learning (Figures 5C–E,I; one-way ANOVA: *F*_(2,19)_ = 1.19376, *p* = 0.324814) compared to controls (*n* = 7) suggesting that this compartment of the POR is not involved in item-place learning.

### Novel Macroscale Item-Place Exploration Results in an Increase in Nuclear Arc mRNA Expression in the Caudal POR

Next, we examined the caudal POR (Figures 5F–H,J). For the caudal POR, microscale item-place learning had no effect on IEG expression in this structure compared to control animals (*n* = 8; one-way ANOVA: *F*_(2,20)_ = 2.4708, *p* = 0.109915). By contrast, the *post hoc* analysis revealed that Arc mRNA levels were significantly changed after macroscale item-place learning compared to control animals (Fisher’s LSD test: *p* = 0.040014).

These results indicate that the PRC and POR are not involved in the processing of microscale visuospatial information. Strikingly, the caudal PRC area 35 and caudal POR only engage in the processing of macroscale item-place information, suggesting that the recruitment of these areas into item encoding may depend on the size of the visuospatial cue.

## Discussion

The present study examined activity-regulated cytoskeletal-associated (Arc) mRNA expression in the POR and PRC following the participation of rats in two kinds of item-place learning tasks. The tasks differed in terms of the size of the cues (large vs. small) and their presentation strategy (overt vs. discrete). We refer to these tasks as macroscale (large, overt) and microscale (small, discrete) item-place learning, respectively. Microscale item-place learning did not affect Arc mRNA expression in the POR (middle and caudal) and areas 35 and 36 of the rostral, middle, and caudal PRC, whereas macroscale item-place learning resulted in an increase in nuclear Arc mRNA expression in the caudal PRC area 35 and the caudal POR. In all other regions that were analyzed, including the caudal PRC area 36, rostral and middle PRC areas 35 and 36, and middle POR, macroscale item-place learning did not affect Arc mRNA expression.

The two types of novel item-place exploration tasks were chosen because they enable synaptic plasticity and trigger nuclear IEG expression in the hippocampus (Kemp and Manahan-Vaughan, 2004, 2008; Hagena and Manahan-Vaughan, 2011; Hoang et al., 2018). Furthermore, a former study demonstrated that 5 min of exposure to either microscale or macroscale cue configurations are sufficient to form memories (Hoang et al., 2018). In both tasks, exchanging one of the cues for a novel cue results in an increase in the exploration of the novel cue, supporting the interpretation that cues and locations are recognized (Hoang et al., 2018). In the present study, the cues within each of the two tasks were explored equally, thus there was no preference for, or avoidance of, single objects. Exploration times of the cues in the microscale and macroscale item-place tasks are comparable to previous results (Hoang et al., 2018).

In the rat brain, the PRC is located along the rhinal sulcus and it is composed of Brodmann areas 35 and 36 (Brodmann, 1909) and the caudally adjacent POR (Burwell, 2001). Area 36 occupies the dorsal bank of the rhinal sulcus and area 35 occupies the ventral bank (Burwell, 2001). Lesion studies have demonstrated that the PRC is involved in object recognition and supports the solving of navigational problems during allocentric spatial learning (Aggleton et al., 1997; Ramos, 2002, 2008, 2013a,b, 2017; Ramos and Vaquero, 2005; Albasser et al., 2009,2011; Albasser et al., 2015). Moreover, the PRC plays an essential role in tactile discrimination tasks in rats (Ramos, 2014a,b, 2016). The PRC can be further subdivided into the rostral, middle, and caudal regions along the anterior-posterior axis (Burwell, 2001; Agster and Burwell, 2009; Albasser et al., 2010, 2013). Information from the neocortex, entorhinal cortices, and temporal cortices terminate heavily in and around layers 1, 2, and 3 (Burwell et al., 1995; Burwell and Amaral, 1998a,b), whereas layer 2/3 and 5 receive input from the insular cortex, cingulate area, somatosensory regions and the visual associational cortex (Burwell and Amaral, 1998a). A recent electrophysiological study has shown that three-dimensional objects can activate neurons in layers 2 and 5 of the PRC (Burke et al., 2012b). Electrophysiological recordings from layer 2/3 of the PRC demonstrate that short- and long-term forms of synaptic plasticity exist in the PRC, which may play a role in the neuronal responses associated with visual recognition memory (Ziakopoulos et al., 1999). In our study, we investigated the involvement of layer 2 of PRC in novel item-place learning. We found, that caudal PRC area 36 and rostral and middle PRC areas 35 and 36 did not respond to macroscale item-place learning, whereas macroscale item-place learning triggered a significant increase in Arc mRNA expression in the caudal PRC area 35.

The caudal PRC, and in particular, area 36 receives strong inputs from the visual associational cortices and temporal areas (Burwell and Amaral, 1998a; Burwell, 2000; Furtak et al., 2007). Others reported that the rostral part of the PRC is mainly involved in recognition memory in darkness (Albasser et al., 2013). By contrast, areas 35 and 36 of the caudal PRC specifically exhibit an important role in identifying object novelty in the presence of light (Albasser et al., 2010). In the present study, the experiment was conducted under illuminated conditions and we observed no changes in Arc mRNA expression in the rostral PRC following the exploration of item-place configurations. This result is in line with the finding from Albasser et al. (2013). Furthermore, our results demonstrate that only area 35 of the caudal PRC is involved in the processing of macroscale item-place learning, thereby being only partially in line with a previous study examining object recognition in light (Albasser et al., 2010). Neuroanatomical and functional connectivity studies have shown that parahippocampal structures such as LEC, MEC, POR, and PRC are highly interconnected (Furtak et al., 2007; Kealy and Commins, 2011). Even though the interconnectivity is not very strong, dorsal hippocampal structures project more strongly to caudal PRC area 35, than to caudal PRC area 36 (Furtak et al., 2007; Agster and Burwell, 2009, 2013). Caudal PRC area 35 receives the strongest inputs from the dorsal CA1 region and subiculum compared to caudal PRC area 36 (Furtak et al., 2007; Kealy and Commins, 2011; Agster and Burwell, 2013). Cortical and subcortical inputs to the caudal PRC support its role in the processing of detailed and multimodal information about objects, including their behavioral relevance, such as familiarity (Burwell and Amaral, 1998a,b; Burwell, 2001; Furtak et al., 2007; Sia and Bourne, 2008). Caudal PRC area 35 receives a moderate projection from the midline thalamic structures, involved in attention, and different aspect of awareness, and the nucleus reuniens, important for crossmodal processing and multimodal sensory information processing (van der Werf et al., 2002; Furtak et al., 2007; Agster et al., 2016). Furthermore, the ventral midline thalamus nuclei are distinctively involved in cue-dependent spatial information processing of CA1 place cells (Jung et al., 2019). Viewed from this perspective, our observation, that the caudal PRC area 35 is involved in macroscale item-place learning, seems logical.

Interestingly, in our study, area 36 of the PRC (rostral to caudal) neither responded to macroscale, nor microscale cue presentation. One study showed that caudal PRC areas 35 and 36 are activated equally when an animal explored novel objects compared to familiar objects (Albasser et al., 2010). In the current study the task differed from the one used by Albasser and colleagues, in that we used two kinds of item sizes and they were presented in spatial constellations. The results of caudal PRC area 35 suggest that attention capture, due to the difference in the size of the items, plays a role in learning (Proulx, 2010). As already mentioned, caudal PRC area 35 receives moderate inputs from thalamic midline nuclei involved in attention (van der Werf et al., 2002; Furtak et al., 2007; Agster et al., 2016). By contrast, PRC area 36 is less strongly connected with hippocampal and some parahippocampal regions e.g., entorhinal cortices, whereas the connections to PRC/POR are stronger, compared to caudal PRC area 35 (Furtak et al., 2007). This difference in anatomical connections could be the reason why both caudal PRC subregions are differently activated after this kind of microscale and macroscale item-place exploration.

For the POR, involvement in the encoding of the spatial arrangement of objects, or context has been suggested (Burwell, 2000; Wang et al., 2018, 2020; Doan et al., 2019; LaChance et al., 2019). Furthermore, the POR may be involved in the processing of non-spatial information (Furtak et al., 2012). Hippocampal, cortical, and subcortical inputs differ across rostral to caudal regions of the POR (Agster and Burwell, 2009; Agster et al., 2016; Tomás Pereira et al., 2016), suggesting a functional differentiation along the rostrocaudal axis of the POR in learning and memory. Tracing studies demonstrate that all rostral to caudal POR regions have efferent and afferent projections to the hippocampus, cortical and subcortical regions, but the heaviest projections are sent and received by the caudal POR (Agster and Burwell, 2009; Agster et al., 2016; Tomás Pereira et al., 2016). Subcortical inputs to caudal POR are dominated by the dorsal thalamus, especially by the lateral posterior nucleus (Tomás Pereira et al., 2016), suggesting the involvement of caudal POR in visuospatial attention (Posner and Petersen, 1990; Shipp, 2004). This implies a dominance of caudal POR over middle and rostral POR in visuospatial information processing. Our results from the present study suggest that the middle and caudal POR do not respond to microscale item-place learning, whereas solely the caudal POR is involved in the encoding of macroscale item-place information.

Several studies suggest that hippocampal subregions, comprising the distal CA1 region (close to the subiculum) and the proximal CA3 region (close to the DG) preferentially process an item’s features (“what” stream), whereas the proximal CA1 region and the distal CA3 region (both close to the CA2 region) preferentially process spatial information (“where” stream; Amaral and Witter, 1989; Witter et al., 1989, 2000; Witter, 2007; Sauvage et al., 2013; Hoang et al., 2018). According to the two-streams hypothesis, the POR and MEC are part of the “where” stream (Burwell, 2000; Hargreaves et al., 2005; Eichenbaum and Lipton, 2008; Eichenbaum et al., 2012), whereas the “what” stream is proposed to include the PRC and LEC (Burwell, 2000; Hargreaves et al., 2005; Eichenbaum and Lipton, 2008). We previously provided evidence that microscale item-place configurations may contain a stronger “what” informational component, compared to “where” information (Hoang et al., 2018). In that study, microscale item-place exploration induces an increase in IEG expression in the distal CA1 region (belonging to the “what” stream), but not in the proximal CA1 region (associated with the “where” stream) and also induces an increase in IEG expression in the proximal (“what” stream) but not the distal CA3 region (“where” stream; Burwell, 2000; Hargreaves et al., 2005; Eichenbaum and Lipton, 2008; Hoang et al., 2018). Information from the POR converges on hippocampal regions of the CA1 and subiculum, but more importantly, the POR has a stronger influence on the proximal CA1 region that is associated with the “where” stream (Burwell and Amaral, 1998a,b; Agster and Burwell, 2009). Together with the results from Hoang et al. (2018), our results confirm microscale item-place configurations have a stronger “what” component than “where” component. This suggests, in turn, that this form of learning did not meet the criteria required for the recruitment of the POR.

Nonetheless, several studies have contradicted the clear differentiation of the two-stream hypothesis, and indicate that the POR is also involved in non-spatial information processing (Furtak et al., 2012; Heimer-McGinn et al., 2017; Burke et al., 2018). Anatomical data suggest the caudal POR is more heavily connected with the visual associational cortex and dorsal thalamus than middle POR (Agster and Burwell, 2009; Agster et al., 2016; Tomás Pereira et al., 2016). Another study examining the POR supports a role for this structure in visual object discrimination (Furtak et al., 2012). The POR projects to both entorhinal cortices and a recent electrophysiological study suggests that LEC layer 2 receives input from POR and projections towards the MEC from POR are less prominent than previously suggested (Burwell and Amaral, 1998a,b; Doan et al., 2019). Furthermore, the POR is believed to receive information about items from the PRC to integrate a contextual representation (Heimer-McGinn et al., 2017; Park et al., 2017; Burke et al., 2018). Thus, the POR is involved not only in the encoding of egocentric and allocentric spatial maps (LaChance et al., 2019; Wang et al., 2020), but also in multi-sensory information processing (Doan et al., 2019). As stated above, we previously reported that macroscale item-place configurations induce an increase in IEG expression in the proximal (“what” stream) but not the distal CA3 region (“where” stream; Burwell, 2000; Hargreaves et al., 2005; Eichenbaum and Lipton, 2008; Hoang et al., 2018). This finding suggests that a strong “what” component is a feature of learning about macroscale item-place configurations (Hoang et al., 2018). As supported by anatomical and neurophysiological studies, the caudal POR seems to be involved in “what” information processing (Burwell and Amaral, 1998a; Agster and Burwell, 2009; Furtak et al., 2012). Here, we detected a significant elevation of Arc mRNA expression after macroscale cue exploration in the caudal POR, but not in the middle POR. In line with this, it has been reported that the POR can be recruited into spatial information processing when spatial navigation is a major component of the generation of spatial representation (Burwell and Hafeman, 2003; Heimer-McGinn et al., 2017; Park et al., 2017), even though this is an aspect that may have played a subordinate role in the present study. Nevertheless, the caudal POR could play a role in spatial perception during navigation of the macroscale item-place configuration in the present study, and even more likely, in item-place mnemonics in a spatial context.

Even though the PRC and POR have been reported to be involved in the recognition of objects in context (Burke et al., 2012b; Furtak et al., 2012; Barker and Warburton, 2015; Heimer-McGinn et al., 2017; Hernandez et al., 2017; Park et al., 2017), in our study we observed that the PRC and POR failed to respond to *microscale* item-place learning. These results are striking, given that higher levels of rears occurred during microscale compared to macroscale item-place exploration, suggesting that the former type of item-place learning required a greater degree of effort and recruited higher levels of attention. By contrast, the exploration time for each of the microscale cues was shorter than the times for the large, overt objects during macroscale item-place learning. These shorter exploration times of the microscale cues could lead to the assumption that the item-place locations were less effectively encoded. However, a comparison of item exploration times with a former study using the same item-place tasks to trigger hippocampal IEG encoding (Hoang et al., 2018) can directly vitiate this conclusion: the exploration times of microscale cues were similar in the present and the previous and thus, were likely to have been sufficient for the encoding of item-place locations. Microscale cues are smaller and more discrete compared to the macroscale cues that are large and overt. A large object can indeed capture attention in a stimulus-driven manner if one is strongly engaging in orientation in space, compared to a smaller object (Proulx, 2010). In our present study, microscale cues hidden in the holes did not extend above the floor level and could not be seen until the animals approached the holes and explored the cues. Under these circumstances, we detected no changes in Arc mRNA expression in PRC and POR. This experimental design contrasts with approaches used by other scientists where objects were visible from afar and where PRC and POR activation was triggered (Furtak et al., 2012; Barker and Warburton, 2015; Heimer-McGinn et al., 2017; Hernandez et al., 2017; Park et al., 2017; Ramos, 2017). This latter strategy enables that the features of the objects can be detected without a close approach and permits that the animals can predict and interpret their positions respective to the objects. This may explain why both the PRC and POR could be activated in those experiments. Taking this into account, our findings suggest that the size of an item and how the item is presented in a given context can determine the extent of information processing in the subregions of the PRC and POR.

## Conclusions

In this study, we explored whether microscale and macroscale item-place learning triggers nuclear mRNA expression of the IEG Arc in the middle and caudal POR as well as in the rostral, middle, and caudal PRC areas 35 and 36. We observed that Arc mRNA expression was unchanged in the middle POR, caudal PRC area 36, and rostral and middle PRC areas 35 and 36 by either form of item-place learning. By contrast, macroscale item-place exploration, but not microscale item-place exploration, induces an increase in IEG expression in the caudal POR, but not middle POR, supporting a role of the caudal POR in item-place learning. Similarly, the caudal PRC exhibited an increase of nuclear IEG expression in area 35, but not area 36, in response to macroscale item-place, but not microscale item-place learning, supporting involvement of the caudal, but not rostral and middle RPC in the encoding of macroscale visuospatial information. This suggests that item dimensions may play a role in the engagement of the PRC and POR in item-place processing. Moreover, our data support that a functional differentiation between areas 35 and 36 of the rostral, middle and caudal PRC, as well as middle and caudal POR, exists in information processing concerning the encoding of item-place configurations.

## Data Availability Statement

The raw data supporting the conclusions of this article will be made available by the authors, without undue reservation.

## Ethics Statement

The animal study was reviewed and approved by Landesamt für Arbeitsschutz, Naturschutz, Umweltschutz und Verbraucherschutz, Northrhine Westphalia, Germany.

## Author Contributions

DM-V devised the concept and experimental strategy of the study. Experiments and data analysis were conducted by NS, CS, and T-HH. DM-V wrote the article, with contributions from all authors.

## Conflict of Interest

The authors declare that the research was conducted in the absence of any commercial or financial relationships that could be construed as a potential conflict of interest.

## References

[B1] Abraham W. C. Mason S. E. Demmer J. Williams J. M. Richardson C. L. Tate W. P.. (1993). Correlations between immediate early gene induction and the persistence of long-term potentiation. Neuroscience 56, 717–727. 10.1016/0306-4522(93)90369-q 8255430

[B5] Aggleton J. P. Keen S. Warburton E. C. Bussey T. J. (1997). Extensive cytotoxic lesions involving both the rhinal cortices and area TE impair recognition but spare spatial alternation in the rat. Brain Res. Bull. 43, 279–287. 10.1016/s0361-9230(97)00007-5 9227838

[B6] Aggleton J. P. Kyd R. J. Bilkey D. K. (2004). When is the perirhinal cortex necessary for the performance of spatial memory tasks? Neurosci. Biobehav. Rev. 28, 611–624. 10.1016/j.neubiorev.2004.08.007 15527866

[B7] Agster K. L. Burwell R. D. (2009). Cortical efferents of the perirhinal, postrhinal, and entorhinal cortices of the rat. Hippocampus 19, 1159–1186. 10.1002/hipo.20578 19360714 PMC3066185

[B8] Agster K. L. Burwell R. D. (2013). Hippocampal and subicular efferents and afferents of the perirhinal, postrhinal, and entorhinal cortices of the rat. Behav. Brain Res. 254, 50–64. 10.1016/j.bbr.2013.07.005 23872326 PMC3792719

[B9] Agster K. L. Tomás Pereira I. Saddoris M. P. Burwell R. D. (2016). Subcortical connections of the perirhinal, postrhinal, and entorhinal cortices of the rat. II. efferents. Hippocampus 26, 1213–1230. 10.1002/hipo.22600 27101786 PMC5070461

[B10] Albasser M. M. Amin E. Iordanova M. D. Brown M. W. Pearce J. M. Aggleton J. P. (2011). Perirhinal cortex lesions uncover subsidiary systems in the rat for the detection of novel and familiar objects. Eur. J. Neurosci. 34, 331–342. 10.1111/j.1460-9568.2011.07755.x 21707792 PMC3170480

[B11] Albasser M. M. Davies M. Futter J. E. Aggleton J. P. (2009). Magnitude of the object recognition deficit associated with perirhinal cortex damage in rats. Effects of varying the lesion extent and the duration of the sample period. Behav. Neurosci. 123, 115–124. 10.1037/a0013829 19170436

[B12] Albasser M. M. Olarte-Sánchez C. M. Amin E. Brown M. W. Kinnavane L. Aggleton J. P. (2015). Perirhinal cortex lesions in rats. Novelty detection and sensitivity to interference. Behav. Neurosci. 129, 227–243. 10.1037/bne0000049 26030425 PMC4450885

[B13] Albasser M. M. Olarte-Sánchez C. M. Amin E. Horne M. R. Newton M. J. Warburton E. C.. (2013). The neural basis of nonvisual object recognition memory in the rat. Behav. Neurosci. 127, 70–85. 10.1037/a0031216 23244291 PMC3569044

[B14] Albasser M. M. Poirier G. L. Aggleton J. P. (2010). Qualitatively different modes of perirhinal-hippocampal engagement when rats explore novel vs. familiar objects as revealed by c-Fos imaging. Eur. J. Neurosci. 31, 134–147. 10.1111/j.1460-9568.2009.07042.x 20092559 PMC4235254

[B15] Amaral D. G. Witter M. P. (1989). The three-dimensional organization of the hippocampal formation. A review of anatomical data. Neuroscience 31, 571–591. 10.1016/0306-4522(89)90424-7 2687721

[B16] André M. A. E. Manahan-Vaughan D. (2013). Spatial olfactory learning facilitates long-term depression in the hippocampus. Hippocampus 23, 963–968. 10.1002/hipo.22158 23804412

[B17] Barker G. R. I. Warburton E. C. (2015). Object-in-place associative recognition memory depends on glutamate receptor neurotransmission within two defined hippocampal-cortical circuits. A critical role for AMPA and NMDA receptors in the hippocampus, perirhinal, and prefrontal cortices. Cereb. Cortex 25, 472–481. 10.1093/cercor/bht245 24035904 PMC4380082

[B18] Brakeman P. R. Lanahan A. A. O’Brien R. Roche K. Barnes C. A. Huganir R. L.. (1997). Homer: a protein that selectively binds metabotropic glutamate receptors. Nature 386, 284–288. 10.1038/386284a0 9069287

[B19] Brodmann K. (1909). Vergleichende Lokalisationslehre der Grosshirnrinde in ihren Prinzipien dargestellt auf Grund des Zellenbaues. Barth JA: Leipzig.

[B20] Brown M. W. Aggleton J. P. (2001). Recognition memory: what are the roles of the perirhinal cortex and hippocampus? Nat. Rev. Neurosci. 2, 51–61. 10.1038/35049064 11253359

[B21] Burke S. N. Gaynor L. S. Barnes C. A. Bauer R. M. Bizon J. L. Roberson E. D.. (2018). Shared functions of perirhinal and parahippocampal cortices: implications for cognitive aging. Trends Neurosci. 41, 349–359. 10.1016/j.tins.2018.03.001 29555181 PMC5970964

[B23] Burke S. N. Maurer A. P. Hartzell A. L. Nematollahi S. Uprety A. Wallace J. L.. (2012b). Representation of three-dimensional objects by the rat perirhinal cortex. Hippocampus 22, 2032–2044. 10.1002/hipo.22060 22987680 PMC3447635

[B24] Burwell R. D. (2000). The parahippocampal region: corticocortical connectivity. Ann. N Y Acad. Sci. 911, 25–42. 10.1111/j.1749-6632.2000.tb06717.x 10911865

[B25] Burwell R. D. (2001). Borders and cytoarchitecture of the perirhinal and postrhinal cortices in the rat. J. Comp. Neurol. 437, 17–41. 10.1002/cne.1267 11477594

[B26] Burwell R. D. Amaral D. G. (1998a). Cortical afferents of the perirhinal, postrhinal, and entorhinal cortices of the rat. J. Comp. Neurol. 398, 179–205. 10.1002/(sici)1096-9861(19980824)398:2<179::aid-cne3>3.0.co;2-y 9700566

[B27] Burwell R. D. Amaral D. G. (1998b). Perirhinal and postrhinal cortices of the rat: Interconnectivity and connections with the entorhinal cortex. J. Comp. Neurol. 391, 293–321. 10.1002/(sici)1096-9861(19980216)391:3<293::aid-cne2>3.0.co;2-x 9492202

[B29] Burwell R. D. Bucci D. J. Sanborn M. R. Jutras M. J. (2004). Perirhinal and postrhinal contributions to remote memory for context. J. Neurosci. 24, 11023–11028. 10.1523/JNEUROSCI.3781-04.2004 15590918 PMC6730280

[B28] Burwell R. D. Hafeman D. M. (2003). Positional firing properties of postrhinal cortex neurons. Neuroscience 119, 577–588. 10.1016/s0306-4522(03)00160-x 12770570

[B30] Burwell R. D. Witter M. P. Amaral D. G. (1995). Perirhinal and postrhinal cortices of the rat: a review of the neuroanatomical literature and comparison with findings from the monkey brain. Hippocampus 5, 390–408. 10.1002/hipo.450050503 8773253

[B31] Bussey T. J. Duck J. Muir J. L. Aggleton J. P. (2000). Distinct patterns of behavioral impairments resulting from fornix transection or neurotoxic lesions of the perirhinal and postrhinal cortices in the rat. Behav. Brain Res. 111, 187–202. 10.1016/s0166-4328(00)00155-8 10840144

[B32] Bussey T. J. Muir J. L. Aggleton J. P. (1999). Functionally dissociating aspects of event memory: the effects of combined perirhinal and postrhinal cortex lesions on object and place memory in the rat. J. Neurosci. 19, 495–502. 10.1523/JNEUROSCI.19-01-00495.1999 9870977 PMC6782353

[B33] Deshmukh S. S. Johnson J. L. Knierim J. J. (2012). Perirhinal cortex represents nonspatial, but not spatial, information in rats foraging in the presence of objects: comparison with lateral entorhinal cortex. Hippocampus 22, 2045–2058. 10.1002/hipo.22046 22987681 PMC3870144

[B34] Dietz B. Manahan-Vaughan D. (2017). Hippocampal long-term depression is facilitated by the acquisition and updating of memory of spatial auditory content and requires mGlu5 activation. Neuropharmacology 115, 30–41. 10.1016/j.neuropharm.2016.02.026 27055771

[B35] Doan T. P. Lagartos-Donate M. J. Nilssen E. S. Ohara S. Witter M. P. (2019). Convergent projections from perirhinal and postrhinal cortices suggest a multisensory nature of lateral, but not medial, entorhinal cortex. Cell Rep. 29, 617.e7–627.e7. 10.1016/j.celrep.2019.09.005 31618631

[B37] Eacott M. J. Gaffan D. Murray E. A. (1994). Preserved recognition memory for small sets, and impaired stimulus identification for large sets, following rhinal cortex ablations in monkeys. Eur. J. Neurosci. 6, 1466–1478. 10.1111/j.1460-9568.1994.tb01008.x 8000570

[B38] Eacott M. J. Machin P. E. Gaffan E. A. (2001). Elemental and configural visual discrimination learning following lesions to perirhinal cortex in the rat. Behav. Brain Res. 124, 55–70. 10.1016/s0166-4328(01)00234-0 11423166

[B36] Eacott M. J. Norman G. (2004). Integrated memory for object, place, and context in rats: a possible model of episodic-like memory? J. Neurosci. 24, 1948–1953. 10.1523/JNEUROSCI.2975-03.2004 14985436 PMC6730393

[B39] Eichenbaum H. Lipton P. A. (2008). Towards a functional organization of the medial temporal lobe memory system. Role of the parahippocampal and medial entorhinal cortical areas. Hippocampus 18, 1314–1324. 10.1002/hipo.20500 19021265 PMC2592493

[B40] Eichenbaum H. Sauvage M. Fortin N. Komorowski R. Lipton P. (2012). Towards a functional organization of episodic memory in the medial temporal lobe. Neurosci. Biobehav. Rev. 36, 1597–1608. 10.1016/j.neubiorev.2011.07.006 21810443 PMC3227798

[B41] Ekstrom A. D. Arnold A. E. G. F. Iaria G. (2014). A critical review of the allocentric spatial representation and its neural underpinnings: toward a network-based perspective. Front. Hum. Neurosci. 8:803. 10.3389/fnhum.2014.00803 25346679 PMC4193251

[B42] Furtak S. C. Ahmed O. J. Burwell R. D. (2012). Single neuron activity and theta modulation in postrhinal cortex during visual object discrimination. Neuron 76, 976–988. 10.1016/j.neuron.2012.10.039 23217745 PMC3523310

[B43] Furtak S. C. Wei S.-M. Agster K. L. Burwell R. D. (2007). Functional neuroanatomy of the parahippocampal region in the rat: the perirhinal and postrhinal cortices. Hippocampus 17, 709–722. 10.1002/hipo.20314 17604355

[B44] Futter J. E. Davies M. Bilkey D. K. Aggleton J. P. (2006). The effects of cytotoxic perirhinal cortex lesions on spatial learning by rats: a comparison of the dark agouti and Sprague-Dawley strains. Behav. Neurosci. 120, 150–161. 10.1037/0735-7044.120.1.150 16492125

[B45] Guzowski J. F. McNaughton B. L. Barnes C. A. Worley P. F. (1999). Environment-specific expression of the immediate-early gene Arc in hippocampal neuronal ensembles. Nat. Neurosci. 2, 1120–1124. 10.1038/16046 10570490

[B46] Hagena H. Manahan-Vaughan D. (2011). Learning-facilitated synaptic plasticity at CA3 mossy fiber and commissural-associational synapses reveals different roles in information processing. Cereb. Cortex 21, 2442–2449. 10.1093/cercor/bhq271 21493717 PMC3183418

[B47] Hargreaves E. L. Rao G. Lee I. Knierim J. J. (2005). Major dissociation between medial and lateral entorhinal input to dorsal hippocampus. Science 308, 1792–1794. 10.1126/science.1110449 15961670

[B48] Heimer-McGinn V. R. Poeta D. L. Aghi K. Udawatta M. Burwell R. D. (2017). Disconnection of the perirhinal and postrhinal cortices impairs recognition of objects in context but not contextual fear conditioning. J. Neurosci. 37, 4819–4829. 10.1523/JNEUROSCI.0254-17.2017 28411272 PMC5426571

[B49] Hernandez A. R. Reasor J. E. Truckenbrod L. M. Lubke K. N. Johnson S. A. Bizon J. L.. (2017). Medial prefrontal-perirhinal cortical communication is necessary for flexible response selection. Neurobiol. Learn. Mem. 137, 36–47. 10.1016/j.nlm.2016.10.012 27815215 PMC5214530

[B50] Hess U. S. Lynch G. Gall C. M. (1995). Regional patterns of c-fos mRNA expression in rat hippocampus following exploration of a novel environment versus performance of a well-learned discrimination. J. Neurosci. 15, 7796–7809. 10.1523/JNEUROSCI.15-12-07796.1995 8613720 PMC6577969

[B51] Hoang T.-H. Aliane V. Manahan-Vaughan D. (2018). Novel encoding and updating of positional, or directional, spatial cues are processed by distinct hippocampal subfields: evidence for parallel information processing and the “what” stream. Hippocampus 28, 315–326. 10.1002/hipo.22833 29394518 PMC5947642

[B53] Jung D. Huh Y. Cho J. (2019). The ventral midline thalamus mediates hippocampal spatial information processes upon spatial cue changes. J. Neurosci. 39, 2276–2290. 10.1523/JNEUROSCI.2127-18.2019 30659088 PMC6433769

[B54] Kealy J. Commins S. (2011). The rat perirhinal cortex: a review of anatomy, physiology, plasticity, and function. Prog. Neurobiol. 93, 522–548. 10.1016/j.pneurobio.2011.03.002 21420466

[B55] Kelly J. W. McNamara T. P. Bodenheimer B. Carr T. H. Rieser J. J. (2008). The shape of human navigation. How environmental geometry is used in maintenance of spatial orientation. Cognition 109, 281–286. 10.1016/j.cognition.2008.09.001 18952206 PMC2612041

[B56] Kemp A. Manahan-Vaughan D. (2004). Hippocampal long-term depression and long-term potentiation encode different aspects of novelty acquisition. Proc. Natl. Acad. Sci. U S A 101, 8192–8197. 10.1073/pnas.0402650101 15150407 PMC419579

[B57] Kemp A. Manahan-Vaughan D. (2008). The hippocampal CA1 region and dentate gyrus differentiate between environmental and spatial feature encoding through long-term depression. Cereb. Cortex 18, 968–977. 10.1093/cercor/bhm136 17702951

[B58] Kerr K. M. Agster K. L. Furtak S. C. Burwell R. D. (2007). Functional neuroanatomy of the parahippocampal region: the lateral and medial entorhinal areas. Hippocampus 17, 697–708. 10.1002/hipo.20315 17607757

[B59] Kinnavane L. Amin E. Olarte-Sánchez C. M. Aggleton J. P. (2016). Detecting and discriminating novel objects: the impact of perirhinal cortex disconnection on hippocampal activity patterns. Hippocampus 26, 1393–1413. 10.1002/hipo.22615 27398938 PMC5082501

[B60] LaChance P. A. Todd T. P. Taube J. S. (2019). A sense of space in postrhinal cortex. Science 365:eaax4192. 10.1126/science.aax4192 31296737 PMC7063980

[B61] Link W. Konietzko U. Kauselmann G. Krug M. Schwanke B. Frey U.. (1995). Somatodendritic expression of an immediate early gene is regulated by synaptic activity. Proc. Natl. Acad. Sci. U S A 92, 5734–5738. 10.1073/pnas.92.12.5734 7777577 PMC41771

[B62] Lyford G. L. Yamagata K. Kaufmann W. E. Barnes C. A. Sanders L. K. Copeland N. G.. (1995). Arc, a growth factor and activity-regulated gene, encodes a novel cytoskeleton-associated protein that is enriched in neuronal dendrites. Neuron 14, 433–445. 10.1016/0896-6273(95)90299-6 7857651

[B63] Maioli S. Gangarossa G. Locchi F. Andrioli A. Bertini G. Rimondini R. (2012). Excitotoxic lesion of the perirhinal cortex impairs spatial working memory in a delayed-alternation task. Behav. Brain Res. 230, 349–354. 10.1016/j.bbr.2012.02.030 22391121

[B64] Manahan-Vaughan D. Braunewell K.-H. (1999). Novelty acquisition is associated with induction of hippocampal long-term depression. Proc. Natl. Acad. Sci. U S A 96, 8739–8744. 10.1073/pnas.96.15.8739 10411945 PMC17586

[B65] Meunier M. Bachevalier J. Mishkin M. Murray E. A. (1993). Effects on visual recognition of combined and separate ablations of the entorhinal and perirhinal cortex in rhesus monkeys. J. Neurosci. 13, 5418–5432. 10.1523/jneurosci.13-12-05418.1993 8254384 PMC6576426

[B66] Murray E. A. Bussey T. J. (1999). Perceptual-mnemonic functions of the perirhinal cortex. Trends Cogn. Sci. 3, 142–151. 10.1016/s1364-6613(99)01303-0 10322468

[B67] Murray E. A. Bussey T. J. Saksida L. M. (2007). Visual perception and memory. A new view of medial temporal lobe function in primates and rodents. Ann. Rev. Neurosci. 30, 99–122. 10.1146/annurev.neuro.29.051605.113046 17417938

[B68] Oliveira V. C. Carrara R. C. V. Simoes D. L. C. Saggioro F. P. Carlotti C. G. Covas D. T.. (2010). Sudan Black B treatment reduces autofluorescence and improves resolution of *in situ* hybridization specific fluorescent signals of brain sections. Histol. Histopathol. 25, 1017–1024. 10.14670/HH-25.1017 20552552

[B69] Park E.-H. Ahn J.-R. Lee I. (2017). Interactions between stimulus and response types are more strongly represented in the entorhinal cortex than in its upstream regions in rats. eLife 6:e32657. 10.7554/elife.32657 29280734 PMC5771666

[B70] Paxinos G. Watson C. (2005). The Rat Brain in Stereotaxic Coordinates. Amsterdam, Boston: Elsevier Academic Press.

[B71] Posner M. I. Petersen S. E. (1990). The attention system of the human brain. Ann. Rev. Neurosci. 13, 25–42. 10.1146/annurev.ne.13.030190.000325 2183676

[B72] Proulx M. J. (2010). Size matters. Large objects capture attention in visual search. PLoS One 5:e15293. 10.1371/journal.pone.0015293 21203454 PMC3009719

[B73] Ramos J. M. J. (2002). The perirhinal cortex and long-term spatial memory in rats. Brain Res. 947, 294–298. 10.1016/s0006-8993(02)03044-5 12176173

[B74] Ramos J. M. J. (2008). Perirhinal cortex lesions produce retrograde amnesia for spatial information in rats. Consolidation or retrieval? Learn. Mem. 15, 587–596. 10.1101/lm.1036308 18685150

[B75] Ramos J. M. J. (2013a). Differential contribution of hippocampus, perirhinal cortex and postrhinal cortex to allocentric spatial memory in the radial maze. Behav. Brain Res. 247, 59–64. 10.1016/j.bbr.2013.03.017 23511252

[B76] Ramos J. M. J. (2013b). Perirhinal cortex lesions produce retrograde but not anterograde amnesia for allocentric spatial information. Within-subjects examination. Behav. Brain Res. 238, 154–159. 10.1016/j.bbr.2012.10.033 23103402

[B77] Ramos J. M. J. (2014a). Essential role of the perirhinal cortex in complex tactual discrimination tasks in rats. Cereb. Cortex 24, 2068–2080. 10.1093/cercor/bht054 23448873

[B78] Ramos J. M. J. (2014b). Perirhinal cortex lesions attenuate stimulus generalization in a tactual discrimination task in rats. Acta Neurobiol. 74, 15–25. 24718040 10.55782/ane-2014-1968

[B79] Ramos J. M. J. (2016). Perirhinal cortex supports tactual discrimination tasks with increasing levels of complexity. Retrograde effect. Neurobiol. Learn. Mem. 131, 121–130. 10.1016/j.nlm.2016.03.018 27021016

[B80] Ramos J. M. J. (2017). Perirhinal cortex involvement in allocentric spatial learning in the rat. Evidence from doubly marked tasks. Hippocampus 27, 507–517. 10.1002/hipo.22707 28100028

[B81] Ramos J. M. J. Vaquero J. M. M. (2005). The perirhinal cortex of the rat is necessary for spatial memory retention long after but not soon after learning. Physiol. Behav. 86, 118–127. 10.1016/j.physbeh.2005.07.004 16098545

[B82] Sauvage M. M. Nakamura N. H. Beer Z. (2013). Mapping memory function in the medial temporal lobe with the immediate-early gene Arc. Behav. Brain Res. 254, 22–33. 10.1016/j.bbr.2013.04.048 23648768

[B83] Schaefer L. H. Schuster D. Schaffer J. (2004). Structured illumination microscopy. Artefact analysis and reduction utilizing a parameter optimization approach. J. Microsc. 216, 165–174. 10.1111/j.0022-2720.2004.01411.x 15516228

[B84] Schindelin J. Arganda-Carreras I. Frise E. Kaynig V. Longair M. Pietzsch T.. (2012). Fiji: an open-source platform for biological-image analysis. Nat. Methods 9, 676–682. 10.1038/nmeth.2019 22743772 PMC3855844

[B85] Shipp S. (2004). The brain circuitry of attention. Trends Cogn. Sci. 8, 223–230. 10.1016/j.tics.2004.03.004 15120681

[B86] Sia Y. Bourne J. A. (2008). The rat temporal association cortical area 2 (Te2) comprises two subdivisions that are visually responsive and develop independently. Neuroscience 156, 118–128. 10.1016/j.neuroscience.2008.07.002 18674594

[B87] Tomás Pereira I. Agster K. L. Burwell R. D. (2016). Subcortical connections of the perirhinal, postrhinal and entorhinal cortices of the rat. I. afferents. Hippocampus 26, 1189–1212. 10.1002/hipo.22603 27119220 PMC5070464

[B88] Ungerleider L. G. Mishkin M. (1982). “Two cortical visual systems,” in Analysis of Visual Behavior, eds Ingle D. J. Goodale M. A. Mansfield R. J. W. (Cambridge, MA: The MIT Press), 549–586.

[B89] van der Werf Y. D. Witter M. P. Groenewegen H. J. (2002). The intralaminar and midline nuclei of the thalamus. Anatomical and functional evidence for participation in processes of arousal and awareness. Brain Res. Rev. 39, 107–140. 10.1016/s0165-0173(02)00181-9 12423763

[B90] Vann S. D. Brown M. W. Erichsen J. T. Aggleton J. P. (2000). Fos imaging reveals differential patterns of hippocampal and parahippocampal subfield activation in rats in response to different spatial memory tests. J. Neurosci. 20, 2711–2718. 10.1523/jneurosci.20-07-02711.2000 10729352 PMC6772240

[B91] Wang C. Chen X. Knierim J. J. (2020). Egocentric and allocentric representations of space in the rodent brain. Curr. Opin. Neurobiol. 60, 12–20. 10.1016/j.conb.2019.11.005 31794917 PMC7080648

[B92] Wang C. Chen X. Lee H. Deshmukh S. S. Yoganarasimha D. Savelli F.. (2018). Egocentric coding of external items in the lateral entorhinal cortex. Science 362, 945–949. 10.1126/science.aau4940 30467169 PMC6261310

[B93] Winters B. D. Forwood S. E. Cowell R. A. Saksida L. M. Bussey T. J. (2004). Double dissociation between the effects of peri-postrhinal cortex and hippocampal lesions on tests of object recognition and spatial memory. Heterogeneity of function within the temporal lobe. J. Neurosci. 24, 5901–5908. 10.1523/jneurosci.1346-04.2004 15229237 PMC6729235

[B94] Witter M. P. (2007). The perforant path. Projections from the entorhinal cortex to the dentate gyrus. Prog. Brain Res. 163, 43–61. 10.1016/s0079-6123(07)63003-9 17765711

[B95] Witter M. P. Doan T. P. Jacobsen B. Nilssen E. S. Ohara S. (2017). Architecture of the entorhinal cortex a review of entorhinal anatomy in rodents with some comparative notes. Front. Syst. Neurosci. 11:46. 10.3389/fnsys.2017.00046 28701931 PMC5488372

[B96] Witter M. P. Groenewegen H. J. Lopes da Silva F. H. Lohman A. H. (1989). Functional organization of the extrinsic and intrinsic circuitry of the parahippocampal region. Prog. Neurobiol. 33, 161–253. 10.1016/0301-0082(89)90009-9 2682783

[B97] Witter M. P. Naber P. A. van Haeften T. Machielsen W. C. Rombouts S. A. Barkhof F.. (2000). Cortico-hippocampal communication by way of parallel parahippocampal-subicular pathways. Hippocampus 10, 398–410. 10.1002/1098-1063(2000)10:4“398::aid-hipo6„3.0.co;2-k 10985279

[B98] Worley P. F. Bhat R. V. Baraban J. M. Erickson C. A. McNaughton B. L. Barnes C. A. (1993). Thresholds for synaptic activation of transcription factors in hippocampus. Correlation with long-term enhancement. J. Neurosci. 13, 4776–4786. 10.1523/jneurosci.13-11-04776.1993 8229198 PMC6576344

[B99] Zhu X. O. Brown M. W. Aggleton J. P. (1995a). Neuronal signalling of information important to visual recognition memory in rat rhinal and neighbouring cortices. Eur. J. Neurosci. 7, 753–765. 10.1111/j.1460-9568.1995.tb00679.x 7620624

[B100] Zhu X. O. Brown M. W. McCabe B. J. Aggleton J. P. (1995b). Effects of the novelty or familiarity of visual stimuli on the expression of the immediate early gene c-fos in rat brain. Neuroscience 69, 821–829. 10.1016/0306-4522(95)00320-i 8596651

[B103] Ziakopoulos Z. Tillett C. W. Brown M. W. Bashir Z. I. (1999). Input-and layer-dependent synaptic plasticity in the rat perirhinal cortex *in vitro*. Neuroscience 92, 459–472. 10.1016/s0306-4522(98)00764-7 10408597

